# Platelet Vesicles Synergetic with Biosynthetic Cellulose Aerogels for Ultra‐Fast Hemostasis and Wound Healing

**DOI:** 10.1002/adhm.202304523

**Published:** 2024-02-28

**Authors:** Ying Wang, Yicheng Guo, Yuqing Liu, Xiaohong Zhao, Yong Huang, Xiaorong Zhang, Xiaohong Hu, Kibret Mequanint, Gaoxing Luo, Malcolm Xing

**Affiliations:** ^1^ Institute of Burn Research, State Key Laboratory of Trauma and Chemical Poisoning, Southwest Hospital the Third Military Medical University (Army Medical University) Chongqing 400038 China; ^2^ Department of Mechanical Engineering University of Manitoba Winnipeg R3T 2N2 Canada; ^3^ Department of Chemical and Biochemical Engineering University of Western Ontario London N6A 5B9 Canada

**Keywords:** fast blood absorption, fibrin formation, flexible ultra‐light bacterial cellulose aerogels, Platelet vesicles, rapid hemostasis

## Abstract

Achieving hemostasis in penetrating and irregular wounds is challenging because the hemostasis factor cannot arrive at the bleeding site, and substantial bleeding will wash away the blood clot. Since the inherently gradual nature of blood clot formation takes time, a physical barrier is needed before blood clot formation. Herein, an ultra‐light and shape memory hemostatic aerogel consisting of oxidized bacterial cellulose (OBC) and platelet extracellular vesicles (pVEs) is reported. The OBC‐pVEs aerogel provides a physical barrier for the bleeding site by self‐expansion, absorbing the liquid from blood to concentrate platelets and clotting factors and accelerating the clot formation by activating platelets and transforming fibrinogen into fibrin. In the rat liver and tail injury models, the blood loss decreases by 73% and 59%, and the bleeding times are reduced by 55% and 62%, respectively. OBC‐pVEs aerogel has also been shown to accelerate wound healing. In conclusion, this work introduces an effective tool for treating deep, non‐compressible, and irregular wounds and offers valuable strategies for trauma bleeding and wound treatment.

## Graphical abstract

Injectable superabsorbent aerogels of bacterial cellulose working with platelet‐derived extracellular vesicles for fast and efficient bleeding management.

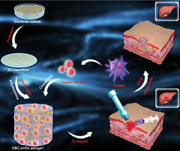

## 1 Introduction

In trauma and surgery, uncontrollable bleeding is a leading cause of death, and rapid hemostasis has clinical significance in saving lives.^[^ ^1^ ^]^ Presently available clinical hemostatic treatments (e.g., gauze compression, gelatin sponges, hemostatic granules, and hemostatic adhesives) have been proven effective in preventing and treating minor hemorrhages.^[^ ^2^, ^3^, ^4^ ^]^ However, more than two million deaths per year are still attributed to uncontrollable bleeding.^[^ ^5^, ^6^ ^]^ For cavitary wounds, sinus tract injuries, and irregular wounds where blood loss is rapid, the hemostatic materials usually do not contact the wound bed well, leading to limited hemostatic efficacy.

Bacterial cellulose (BC) membrane offers good hydrophilicity, biocompatibility, and strong mechanical and eco‐friendly properties but also exhibits slow degradation and inadequate hemostasis.^[^ ^7^ ^]^ Oxidized cellulose (OC), with better hemostatic activity and more efficient biodegradability, has been demonstrated as a good candidate for designing hemostatic materials.^[^ ^8^, ^9^ ^]^ OBC is widely used as a medical dressing owing to its tunable 3D microfiber structures and high specific surface area.^[^ ^10^, ^11^ ^]^ As a result of large porosity and high compressibility, OBC‐based cryogels and aerogels were used for non‐compressible hemorrhage.^[^ ^12^, ^13^, ^14^ ^]^ However, further improvements are required to guarantee the self‐expansion of the injected aerogels, form a physical barrier, and absorb the blood to achieve hemostasis.

Preventing the washout of hemostatic materials and inducing rapid blood coagulation is key to achieving effective hemostasis. Wet resilience and shape recovery behavior of OBC‐based aerogels could ensure wound cavity filling and prevent the hemostatic materials from being washed out while forming a physical barrier. To achieve this goal, 2,2,6,6‐tetramethylpiperidine‐1‐oxyl (TEMPO)‐oxidized BC was treated with ice‐crystal templated self‐assembly to obtain porous structures with high specific surface area and good wet resilience.^[^ ^15^, ^16^ ^]^ In recent years, cellulose aerogels have been reported for hemostasis. For example, cellulose nanofiber/chitosan and carboxymethyl cellulose aerogels possess a good volume expansion ratio and porous structure, which could modulate the coagulation pathway. By fast blood absorption, adhesive/aggregating blood cells (such as platelets and red blood cells), and activation of platelets to accelerate coagulation, cellulose aerogels are demonstrated to be useful hemostatic materials.^[^ ^12^, ^17^, ^18^ ^]^ Notwithstanding this, for severe hemorrhage, the clotting time should be further shortened to achieve effective hemostasis. Introducing bioactive hemostatic components onto the scaffold of cellulose aerogels could, therefore, be a valid approach to enhance the coagulation efficiency.

Extracellular vesicles (EVs) are cell‐derived nanosized discoid vesicles that carry bioactive molecules. It has been reported that EVs are associated with coagulation, inflammation, apoptosis, and angiogenesis.^[^ ^19^ ^]^ Platelets play an important role in physiological hemostasis. The hemostatic function of platelets is achieved through the release of vasoconstrictive substances, the aggregation of platelets to block injured blood vessels, and the promotion of coagulation. Therefore, the potential application for pVEs as the hemostatic agent is considerable. The pVEs could affect multiple pathophysiological roles through intra‐cellular communication, such as coagulation and wound healing, by the ability of EVs to transport proteins, lipids, metabolites, and nucleic acids.^[^ ^20^ ^]^ Many studies have reported that pVEs exhibit good hemostatic effects,^[^ ^21^, ^22^, ^23^ ^]^ while activated platelet‐derived vesicles (Act‐pVEs) are generated as a hemostatic biomaterial because Act‐pVEs possess an active form for αIIbβ3, possibly providing elevated affinity for fibrinogen/fibrin to trigger fibrin clots for hemostasis.^[^ ^24^ ^]^ Taken together, Act‐pVEs are promising hemostatic biomaterial and could further decorate aerogel scaffolds to enhance the hemostasis ability.^[^ ^25^ ^]^

We herein report pVEs‐laden OBC aerogels with shape memory behavior for rapid and active hemostasis in deep or non‐compressible wounds. Mussel‐inspired polydopamine (DA) was used to bind pVEs onto our bacterial cellulose aerogel. Firstly, the OBC was self‐assembled using ice crystal as a template through cyclic freeze‐thaw, obtaining interconnected aerogels with large pores. The aerogels were then coated with DA, and pVEs were adhered to their surface for the hemostatic function of OBC aerogel. When the OBC‐pVEs aerogel was injected into a trauma injury model, it formed a physical barrier on the bleeding site by self‐expansion. Furthermore, it quickly absorbed blood and activated platelets to accelerate and transform fibrinogen into fibrin for rapid hemostasis.

## 2 Result and Discussion

### 2.1 Preparation and Characterization of OBC Aerogels

**Figure 1:**
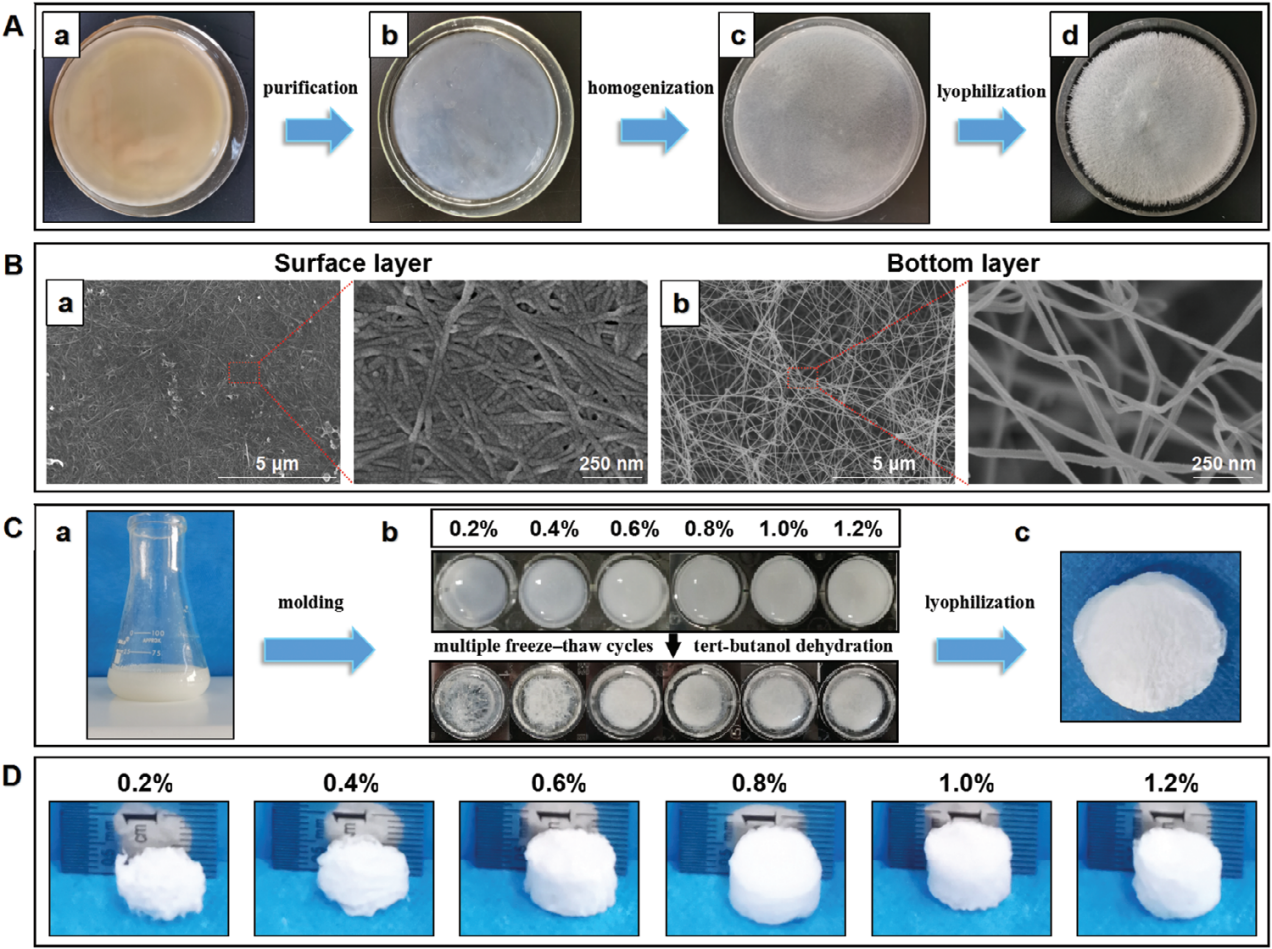
Preparation of OBC aerogels. A) BC membrane digital pictures during the purification, high‐speed homogenization, and lyophilization stages to obtain BC nanofibers. B) SEM images of BC membrane. C) TEMPO oxidation, cyclic freeze‐thaw and tert‐butanol dehydration to prepare OBC aerogels. D) Pictures of 0.2 wt%, 0.4 wt%, 0.6 wt%, 0.8 wt%, 1.0 wt%, and 1.2 wt% OBC aerogels.

As shown in **Figure** **1**A, after purification, high‐speed homogenization, and lyophilization, the obtained BC membrane was turned into BC nanofibers. From the SEM image, the diameter of BC nanofiber membrane was 20–40 nm (Figure 1B). In Figure 1C, the BC nanofibers were first oxidized, and then different concentrations of OBC were subjected to multiple freeze‐thaw cycles and tert‐butanol dehydration to prepare OBC aerogels. OBC was made into a cylindrical shape (Figure 1D). The concentration of OBC aerogels was set at 0.2%, 0.4%, 0.6%, 0.8%, 1.0%, and 1.2%. The 0.2–0.4 wt% OBC aerogels were loose and irregular, whereas, at concentrations >0.6 wt%, the OBC aerogels could be molded into cylindrical shapes that were dimensionally stable. The heights of OBC aerogel cylinders with different concentrations are shown in Figure S1A (Supporting Information). Accordingly, water absorption ratios for 0.2–1.2 wt% OBC aerogels were 11 551.14 ± 103.04, 8252.15 ± 221.60, 6156.56 ± 137.55, 6066.63 ± 73.24, 4377.84 ± 114.53, and 3964.74 ± 67.04 (Figure S1B, Supporting Information). The water absorption for aerogels moderately decreased as the aerogel concentration increased. Mercury intrusion porosimetry showed the porosities of 0.2 wt%, 0.4 wt%, 0.6 wt%, 0.8 wt%, 1.0 wt%,and 1.2 wt % OBC aerogels as 91.91 ± 0.43%, 91.68 ± 1.59%, 93.25 ± 0.70%, 97.35 ± 0.36%, 89.92 ± 2.42% and 92.56 ± 0.45%, respectively. Among them, 0.8 wt% OBC aerogel showed the highest porosity (97.35 ± 0.36%) with a better total pore area (42.22 ± 8.21) m^2^ g^−1^ than other OBC aerogels. Meanwhile, the density of 0.2–1.2 wt% OBC aerogels gradually increased (Table S1, Supporting Information). Therefore, after assessing the morphology, dimensional stability, and water absorption of different OBC content aerogels, 0.8 wt% was chosen as the best candidate for further studies.

### 2.2 Mechanical Properties of OBC Aerogels

**Figure 2:**
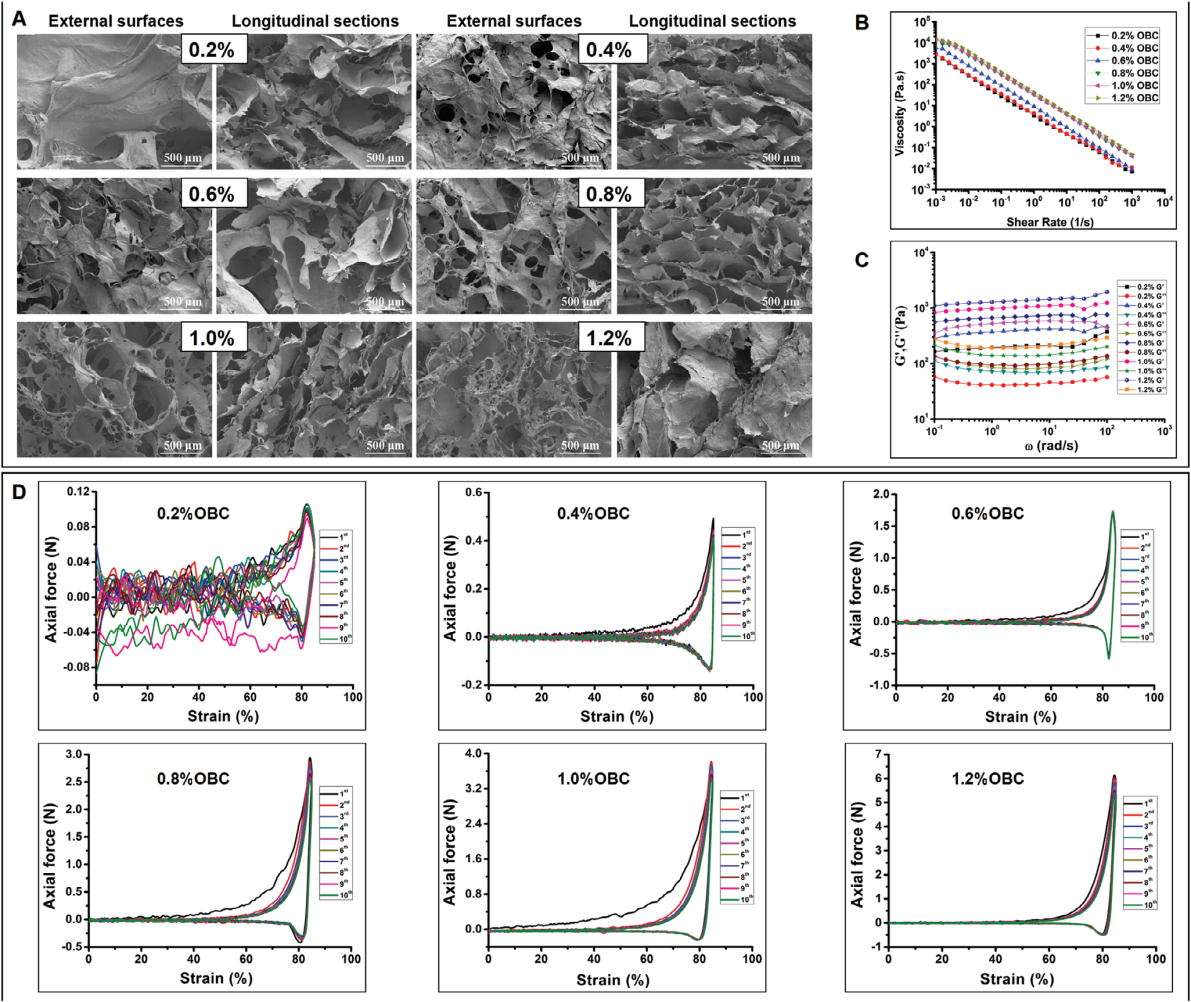
Characterization of OBC aerogels. A) SEM images of 0.2–1.2 wt% OBC aerogels. B) Viscosity (Pa.s) of 0.2–1.2 wt% OBC aerogels at shear rate (1 s^−1^). C) G′ and G″of 0.2–1.2 wt% OBC aerogels upon strain amplitude sweep at a fixed angular frequency (10 rad s^−1^). D) The cyclic compression stress‐train curves of 0.2–1.2 wt% OBC aerogels for 10 cycles at 85% strain.

The SEM images of 0.2–1.2 wt% OBC aerogels (**Figure** **2**A) showed macro‐porous structures, enabling the aerogels to quickly uptake abundant amounts of water. Pore sizes (µm) of 0.2–1.2 wt% OBC aerogels were 28.16 ± 9.58, 11.90 ± 1.49, 7.66 ± 0.38, 6.29 ± 0.31, 4.81 ± 0.41 and 2.56 ± 0.42, shown in Table S1 (Supporting Information). The pore size and shape of these aerogels were related to their concentrations. These results demonstrated that the pores and pore sizes of 0.8 wt% aerogel were more uniform compared to others. Flow properties are affected by viscosity which is a measure of the cohesive forces that exist within the fluid. Figure 2B demonstrates that the as‐prepared OBC aerogels possess non‐Newtonian shear‐thinning viscosities that are appropriate for injection. As shown in Figure 2C, oscillation frequency tests proved that the OBC aerogel was stabilized within the frequency range of 0.1 to 100 rad s^−1^. This stability is useful when the materials are used in tissue filling. A dynamic compression test for 85% strain and 10 cycles provided additional assessment for aerogel mechanical stability. All OBC aerogels possessed low recovery losses and demonstrated acceptable compressive elasticity upon enduring elevated (85%) compressive strain (Figure 2D). This study further evaluated the shape recovery properties and injectability of OBC aerogels (Figure S2 and Movie S1, Supporting Information) and found that 0.2–0.8 wt% OBC aerogels exhibited better shape recovery behavior and were injectable. 1.0 wt% and 1.2 wt% OBC aerogels were easy to break after injection. The wet resilience/injectability can be attributed to the well‐assembled OBC nanofibers resulting from the freezing.^[^ ^26^ ^]^ Overall, we demonstrated that 0.8 wt% OBC aerogels are a suitable candidate for further applications due to the high porosity and shape memory that promote rapid blood absorption and enrichment of coagulation factors.

### 2.3 Characterization of pVEs and OBC‐pVEs Aerogels

**Figure 3:**
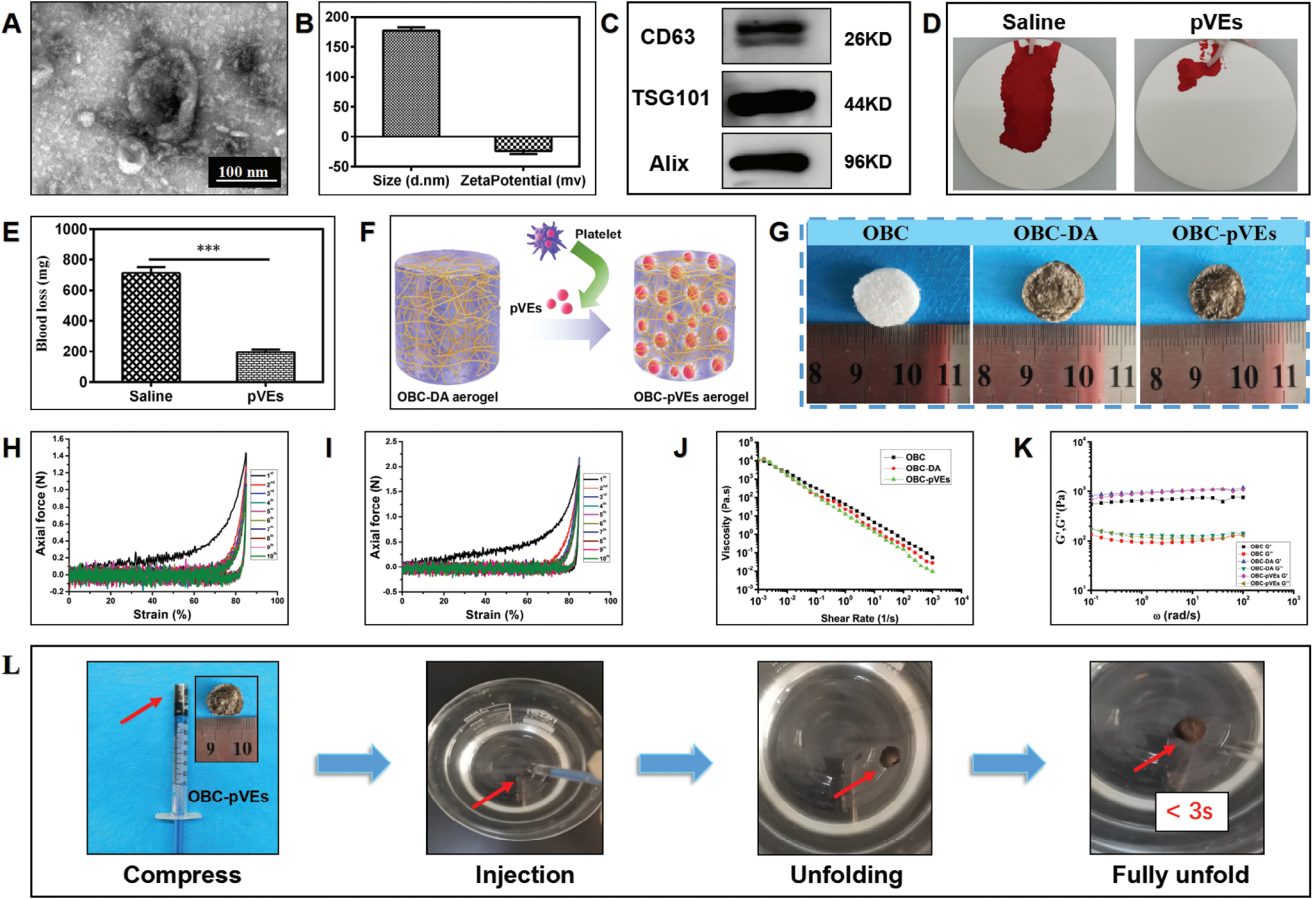
Preparation, characterization, and validation of pVEs and OBC‐pVEs aerogels. A) TEM image of pVEs. B) Particle size and Zeta potential results of pVEs. C) Western blot for the identification of CD63, TSG101, and Alix biomarkers in pVEs. D) Mice tail amputation hemostatic testing. E) Quantification of blood loss. F) Schematic diagram of OBC‐pVEs aerogels fabrication. G) Digital images of OBC, OBC‐DA, and OBC‐pVEs aerogels. H) Cyclic compression stress‐train curves for OBC‐DA aerogel for 10 cycles. I) Cyclic compression stress‐train curves for OBC‐pVEs aerogel for 10 cycles. J) Viscosity (Pa.s) of OBC‐DA and OBC‐pVEs aerogels at sheer rate (1 s^−1^). K) G′and G″of OBC‐DA and OBC‐pVEs aerogels upon strain amplitude sweep with fixed angular frequency (10 rad s^−1^). L) OBC‐pVEs aerogel: Load into 1 mL syringe after wetting, injected from the syringe, sequential images for reabsorbing water to full shape recovery within 3 s. Data in E is presented as means ± S.D. (*n* = 3, Student's *t*‐test, ***p < 0.001).

The characterizations of extracted pVEs, including morphology, particle size, zeta potential, and particle movement, were conducted (**Figures** **3**A,B and S4, Supporting Information). The morphology from the TEM images for pVEs showed that spherical and cup‐shaped pVEs were successfully obtained, while the sizes of pVEs ranged from 30 to 200 nm, which were consistent with previous reports.^[^ ^24^ ^]^ The mean hydrodynamic diameter of pVEs was 177.3 ± 3.1 nm, while the surface charge of pVEs was −23.35 ± 3.11 mV, consistent with TEM analysis. The particle tracking analysis showed that pVEs were well dispersed within the extracts without apparent aggregation and showed good colloidal stability.

Western blotting for CD63 (26 kD), TSG101 (44 kD), and Alix (96 kD) validated the presence of these proteins in pVEs (Figure 3C), indicating successful isolation of pVEs from blood.^[^ ^27^ ^]^ The in vivo hemostatic ability of pVEs was tested using BALB/c tail amputation. After amputation, the tail was immediately placed on an absorbent filter paper (Figure 3D). The pVEs‐treated group had a minor hemorrhage, 196.40 ± 10.15 mg. However, the blood loss of the control group reached 713.47 ± 22.59 mg of blood (Figure 3E). The significant reduction of blood loss within the same period suggested that the pVEs can efficiently stop bleeding. pVEs possess an active site for αIIbβ3, providing elevated affinity for fibrinogen/fibrin to trigger fibrin clots for hemostasis. To ensure the successful use of pVEs for severe bleeding conditions, DA was used to reinforce the 0.8 wt% OBC. After that, we loaded pVEs onto OBC aerogels through mussel‐inspired DA (Figure 3F,G). The mechanical stability of aerogels was evaluated through the dynamic compression‐strain test at 85% strain for 10 cycles. It was found that the compressive force of OBC‐DA and OBC‐pVEs aerogels at the tenth cycle was 1.05 N and 1.98 N, decreasing by less than 20%, compared to the first compression cycle with 1.48 N and 2.16 N. This confirmed that OBC‐DA and OBC‐pVEs aerogels had good mechanical stability (Figure 3H,I and Movie S2, Supporting Information). The viscosity and stability of OBC‐DA and OBC‐pVEs aerogels were unchanged, suggesting that the injectability and stability of OBC‐pVEs aerogels were not negatively affected by loading pVEs and DA (Figure 3J,K). Figure 3L and Movie S3 (Supporting Information) also show that the OBC‐pVEs aerogel has good shape memory behavior and injectability and could fully recover in <3 s.

Cavity wounds, which are often caused by bullets and other major accidents, are life‐threatening due to the uncontrollable bleeding. Therefore, wound packing is a common way to control out‐of‐hospital hemorrhage. Shape memory OBC‐pVEs aerogel could quickly absorb blood, expand to fill the wound site, and form a physical barrier to stop bleeding.

### 2.4 Characterization of OBC‐pVEs Aerogels and In Vitro Biocompatibility Evaluation

**Figure 4:**
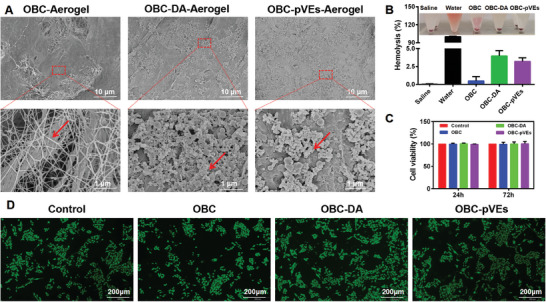
Cytocompatibility and hemocompatibility of aerogels. A) SEM images of OBC, OBC‐DA, and OBC‐pVEs aerogels. B) The hemolysis ratio of aerogels (*n* = 3). C) Cell viability of 3T3 cells using CCK8 test (*n* = 4). D) Fluorescence microscopy imaging for 3T3 cells, or leach medium extract from OBC, OBC‐DA, OBC‐pVEs aerogels after 24 h. In B and C, datasets reflect means ± SD. Bacterial cellulose fiber, DA, and pVEs of OBC, OBC‐DA, and OBC‐pVEs aerogels (red arrow).

SEM was used to evaluate the morphology of aerogels after DA coating and pVEs loading. As shown in **Figure** **4**A, OBC aerogels showed a nanofibrous morphology. After DA deposition, the pVEs adhered to the nanofibers, producing OBC‐pVEs aerogels. The water absorption ratio of OBC‐DA and OBC‐pVEs aerogels were 4062.29 ± 38.78, and 3474.72 ± 228.93 (Figure S3, Supporting Information). From mercury intrusion porosimetry analysis, the porosities of OBC‐DA and OBC‐pVEs aerogels were 93.14 ± 2.61% and 91.46 ± 0.60%, specific pore areas were 2.81 ± 0.27 (m^2^ g^−1^) and 1.79 ± 0.15 (m^2^ g^−1^) which were considerably lower compared with the corresponding OBC‐only aerogels (Table S1, Supporting Information). Their densities were 0.047 ± 0.007 (g mL^−1^) and 0.062 ± 0.011 (g mL^−1^) (Table S2, Supporting Information) and increased by incorporation of DA and pVEs. These results further confirmed that OBC‐pVEs aerogels have a good spatial structure for potential blood absorption. Cell growth and differentiation in a conditioned medium (a medium obtained from incubating the materials with culture media) are important parameters concerning biocompatibility assessment for biomaterials.^[^ ^28^ ^]^ Cellular fluorescence imaging (Figure 4D) presented 3T3 cell morphology after 24 h cultured with the media containing OBC, OBC‐DA, OBC‐pVEs extracts. In all cases, 3T3 cells grew well with normal cell morphology and proliferative properties. CCK‐8 studies demonstrated cellular activities treated with OBC, OBC‐DA, OBC‐pVEs aerogels extracts did not show major variations in comparison with the control group, indicating that each group of aerogels had no toxicity to cells (Figure 4C). Hemolysis risk should be avoided when devices have blood contact applications.^[^ ^29^ ^]^ According to the international ISO10993‐4 standard, a material's acceptable hemolysis rate (HR) is only <5%. The hemolysis results of OBC, OBC‐DA, and OBC‐pVEs aerogels were 0.48% ± 0.35, 3.98% ± 0.43, and 3.20% ± 0.29, respectively, thus meeting the ISO10993‐4 standard (Figure 4B). It indicated that OBC, OBC‐DA, and OBC‐pVEs aerogels exhibited good hemocompatibility for erythrocytes.

### 2.5 Whole Blood Agglutination Test of OBC‐pVEs Aerogels

**Figure 5:**
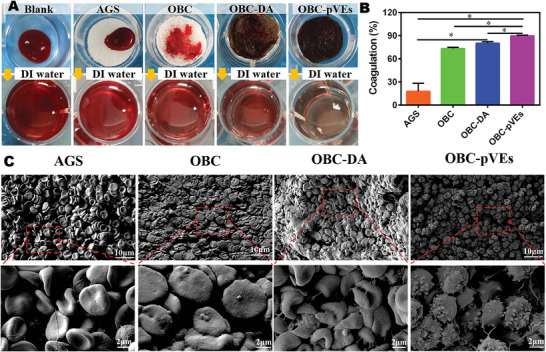
In vitro coagulation assessment. A) In vitro agglutination experiment. B) Invitro blood agglutination percentage on different material surfaces. Data is presented as means ± SD (*n* = 3, One‐way ANOVA, *p < 0.05). C) SEM images for blood components when different materials are exposed to whole blood.

Blood coagulation ability is one of the important indexes to evaluate whether or not a material possesses hemostatic function. As shown in **Figure** **5**A,B and Movie S4 (Supporting Information), hemoglobin release was assessed through whole‐blood agglutination. Within 2 min, the agglutination rate of AGS was 17.89 ± 6.00%. Since anticoagulant blood drops could not be absorbed quickly when added to the surface of AGS, they stayed on the surface and formed a ball, which resulted in a very low agglutination rate in vitro. The agglutination rate of OBC, OBC‐DA, and OBC‐pVEs was 73.26 ± 0.87%, 80.19 ± 1.19%, and 89.76 ± 0.49%, which were considerably elevated in comparison with AGS (*p* = 0.060, *p* = 0.043 and *p* = 0.039). Therefore, OBC, OBC‐DA and OBC‐pVEs possessed excellent coagulation function in vitro. We further investigated the morphology change by SEM when blood contacted the aerogels. For the AGS group, an elevated erythrocyte population was found adhering to aerogels, but only a small amount of fibrin was distributed between red blood cells. However, for the OBC and OBC‐DA groups, the red blood cells were squeezed and warped, with partially activated platelets and limited fibrin between them. In contrast, on the OBC‐pVEs aerogels, abundant activated platelets, some fibrin, and fewer red blood cells bound to the aerogel surface were evident (Figure 5C). These results highlighted that rapid clotting stemmed from OBC‐pVEs, which quickly absorbed blood, activated platelets, and enhanced fibrinogen‐to‐fibrin conversion. Meanwhile, OBC‐pVEs aerogels can considerably lower clotting time, which is a desired feature for hemostatic materials. Although the OBC aerogels alone show that they can rapidly adsorb blood and capture the red blood cells and platelets, the blood clotting time needs to be shortened. The inclusion of dopamine and pVEs in OBC accelerated blood clotting due to active αIIbβ3 on pVEs to provide elevated affinity for fibrinogen/fibrin to trigger the clotting pathway.

### 2.6 In Vivo OBC‐pVEs Aerogel Hemostasis Evaluation

**Figure 6:**
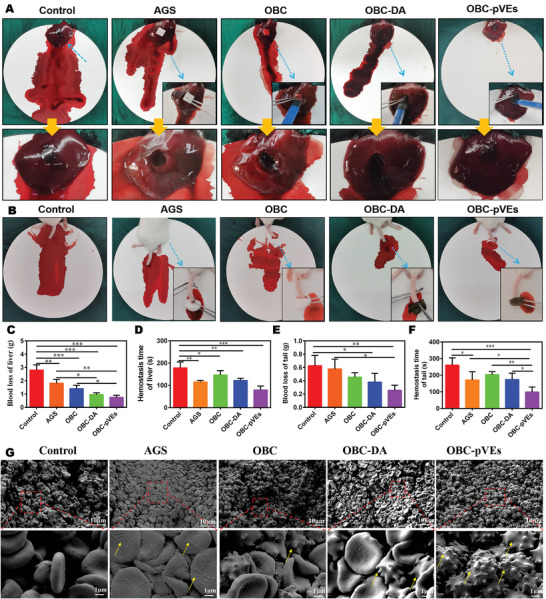
In vivo blood coagulation evaluation of different materials. A) In vivo agglutination process in rat hemorrhaging liver. B) In vivo agglutination process in mice tail amputation model. C–F) Blood loss and hemostasis time for rat liver and mice tail trauma injury models (data presented as means ± SD for *n* = 3, *p < 0.05, **p < 0.01, ***p < 0.001 one‐way ANOVA analysis). G) SEM images for blood clots in the liver (arrows indicate activated platelets and fibrin).

Following in vitro hemostasis evaluation, we next evaluated in vivo hemostatic behavior in a hemorrhaging liver model of rats (**Figure** **6**A and Movie S5, Supporting Information). OBC‐pVEs aerogel was injected into the 6 mm × 3 mm hole on the liver, then self‐expanded to fill the wound to achieve rapid hemostasis. The blood loss and hemostatic time are shown in Figure 6C,D. The wounds treated with OBC‐pVEs showed the lowest blood loss (0.76 ± 0.09 g), while the blood loss for the control reached 2.81 ± 0.22 g. AGS, OBC, OBC‐DA treated wounds showed blood loss of 1.82 ± 0.16 g, 1.41 ± 0.14 g, and 0.96 ± 0.08 g. Blood loss for AGS was significantly lower compared with OBC‐DA (P = 0.02) and OBC‐pVEs (P = 0.0001). The hemostasis time of OBC‐pVEs group (98.67 ± 17.25 s) was considerably shorter than the control (260.67 ± 25.18 s, *p* < 0.001), AGS (171.00 ± 28.62 s, *p* = 0.029), OBC (203.33 ± 10.35 s, *p* = 0.001) and OBC‐DA (174.00 ± 21.93 s, *p* = 0.013) group. Figure 6B shows the hemostasis in the mouse tail amputation model. The blood loss for the control group (0.63 ± 0.09 g) was the highest among all the groups (*p* = 0.032, *p* = 0.004, Figure 6E). AGS, OBC, OBC‐DA, and OBC‐pVEs showed blood loss of 0.58 ± 0.09 g, 0.46 ± 0.04 g, 0.38 ± 0.07 g, and 0.26 ± 0.04 g, while AGS hemorrhaging was significantly elevated compared with OBC‐pVEs group (*p* = 0.029). Meanwhile, the hemostasis time of OBC‐pVEs group (79.67 ± 9.91 s) was considerably less than that for control (178.67 ± 15.19 s, *p* < 0.001), AGS (115.67 ± 3.93 s, *p* = 0.040), OBC (146.67 ± 11.05 s, *p* = 0.007) and OBC‐DA (122.00 ± 5.57 s, *p* = 0.034) group (Figure 6F). In Figure 6G, the hemostatic mechanism is evaluated through direct observation of liver blood clots. The blood clot within the blank group presented plentiful red blood cells, and only limited fibrin meshwork and activated platelets were observed in the OBC and OBC‐DA groups. However, blood clots from the OBC‐pVEs group appeared with irregular multiple protrusions of activated platelets and fibrin meshwork, indicating that rapid hemostasis of OBC‐pVEs aerogel can rapidly activate platelets and promote the conversion of fibrinogen into fibrin meshwork. The significant reduction of hemorrhaging/loss time from current hemostatic aerogels suggested that the OBC‐pVEs aerogels can stop bleeding for efficient hemorrhage control. The possible mechanism of coagulation of OBC‐pVEs aerogels is that the aerogels could lead to rapid physical adsorption and biological interaction. OBC aerogel rapidly absorbs blood, capturing the red cells, platelets, and coagulation factors. As reported before, pVEs with whole membrane proteins of platelets and high surface area to volume ratios have allowed efficient interaction with fibrinogen and other endogenous platelets for successful hemostatic activity in vitro and in vivo.^[^ ^24^ ^]^ Active αIIbβ3 on the surfaces of the pVEs plays a crucial role in hemostasis. After incubation with fibrinogen, it could form dense fibrin networks to enhance hemostasis. On the other hand, sphingosine‐1‐phosphate derived from pVEs could promote wound healing via the S1PR1/AKT/FN1 signaling pathway.^[^ ^30^ ^]^

### 2.7 In Vivo Biocompatibility of OBC‐pVEs Aerogels

**Figure 7:**
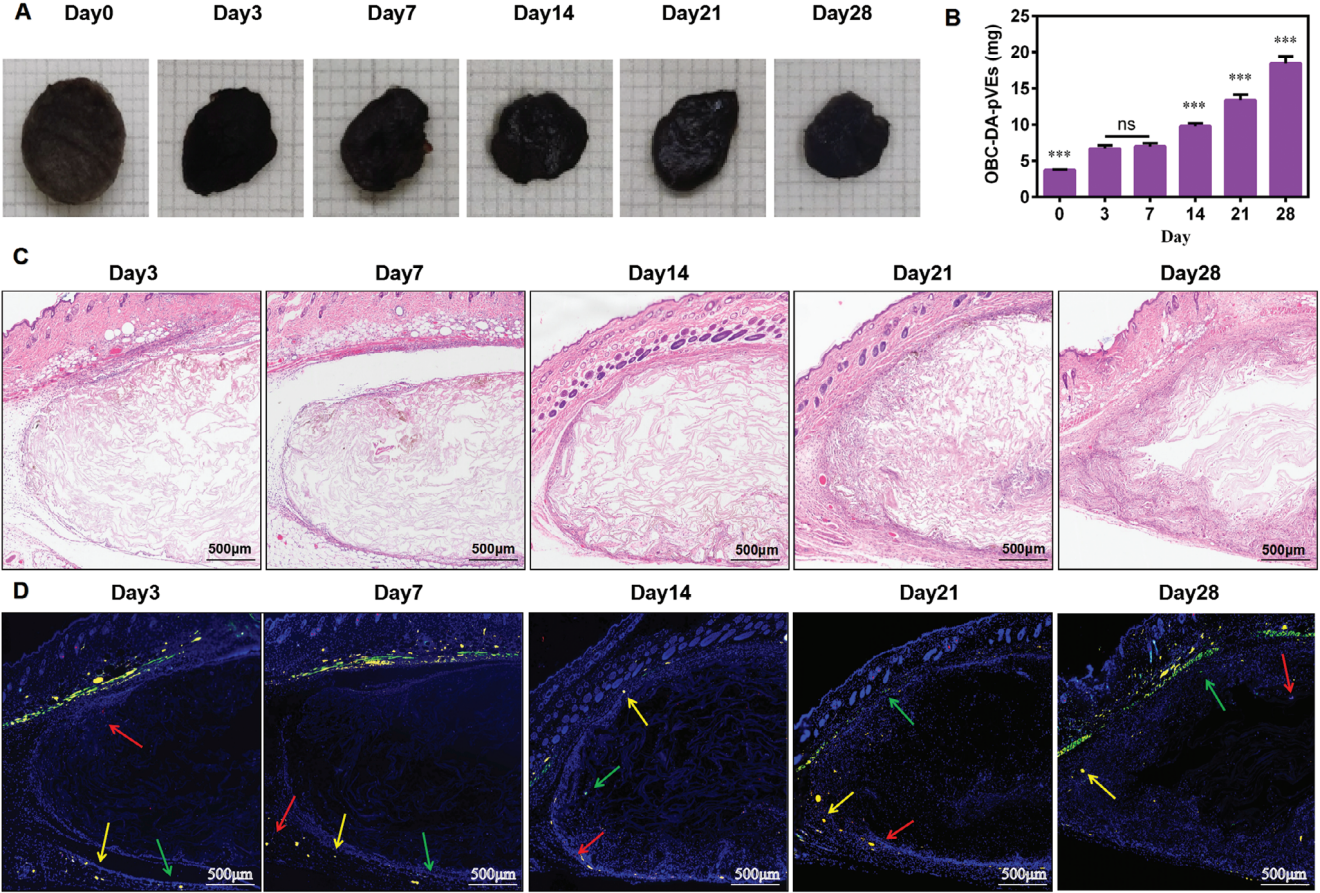
In vivo biocompatibility and degradation of OBC‐pVEs aerogel following subcutaneous implantation in rats. A) Representative digital images of OBC‐pVEs aerogel implants. B) In vivo OBC‐pVEs aerogel weight change for different subcutaneous implantation times (means ± SD for *n* = 3, one‐way ANOVA, ns: *p*>0.05, ***p < 0.001). C) H&E staining of subcutaneous tissues for the indicated times. D) Immunostaining for subcutaneously implanted OBC‐pVEs aerogel for macrophage (F4/80, green fluorescence) and lymphocyte infiltration (CD3, red fluorescence), nuclei (DAPI, blue fluorescence), and the merged color was yellow.

As shown in **Figure** **7**A,B, the volume of the implanted OBC‐pVEs aerogel decreased over time, as indicated by the digital images; however, the weight increased with time, very likely attributed to cellular infiltration and growth as the aerogel was remodeled with densely compacted cellular and extracellular components. H&E staining revealed traces of mononuclear inflammatory cell recruitment (days 3 and 7), suggesting reduced local host inflammatory reaction (Figure 7C). Furthermore, immunofluorescence imaging of subcutaneously implanted OBC‐pVEs aerogel for macrophages (F4/80)/lymphocytes (CD3) showed minimal lymphocyte and macrophage infiltration from day 3 to 28, suggesting limited immune reaction (Figure 7D). These results demonstrated that the OBC‐pVEs aerogel is suitable for cell growth and tissue healing while eliciting minimal inflammatory reactions.

### 2.8 The Effects of OBC‐pVEs Aerogels on wound Healing and wound Re‐Epithelialization

**Figure 8:**
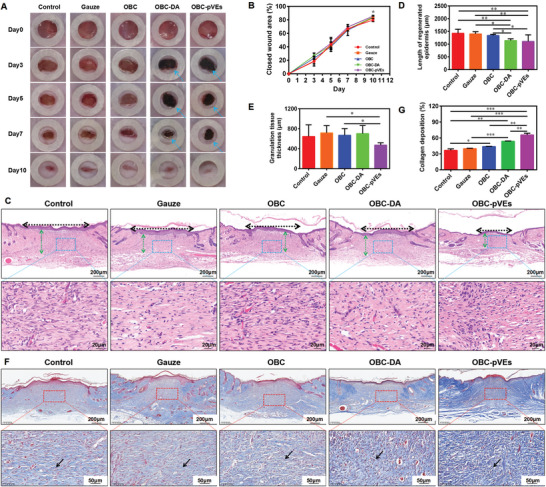
Wound healing evaluation in rat model. A) Macroscopic wound closure images at differing time points with the 6‐mm‐diameter standard silicone ring. B) Percentage of the closed wound area for different treatments. C) H&E staining for wound tissues from individual groups 10 days post‐surgery. D) Quantitative assessment for length of regenerated epidermis 10 days post‐surgery. E) Quantitative assessment for granulation tissue growth 10 days post‐surgery. F) Masson staining of the wound area for collagen deposition on day 10. G) Quantitative analysis of collagen deposition for each group. In (B, D, E, G), datasets reflect means ± SD (*n* = 5, One‐way ANOVA, *p < 0.05, **p < 0.01, ***p < 0.001). The dashed black arrows represent the length of the regenerated epidermis, and the green dotted arrows represent the new granulation thickness. The black arrows in panel (C) are collagen deposition, and the blue arrows in panel (F) indicate the aerogels attached to the wound.

As shown in **Figure** **8**A,B, OBC‐pVEs aerogels considerably accelerated wound healing in a mice model over a period of 10 days. The rate of wound closure was determined by quantifying the area. By day 3, the closed wound area was 26.68 ± 1.75% for OBC‐pVEs aerogel group, while wound closed wound areas were 18.20 ± 1.55%, 18.84 ± 3.25%, 23.05 ± 0.91%, and 26.59 ± 1.86% for control, Gauze, OBC, and OBC‐DA groups, respectively. At days 5 and 7, closed wound areas varied from 40% to 65% for the different groups, and OBC‐pVEs showed the highest closed area of these. After 10 days, OBC‐pVEs reached 85.56 ± 0.51% reduction, whereas control, Gauze, OBC, and OBC‐DA groups reached 79.75 ± 1.11%, 82.72 ± 1.14%, 82.96 ± 0.17%, and 84.08 ± 1.17%, respectively. Consequently, the administration of OBC‐pVEs aerogel considerably accelerated wound healing compared with the control group at 10‐day post‐surgery (*p* = 0.036). OBC‐pVEs aerogel promoted proliferative and migrative properties for keratinocytes, leading to enhanced re‐epithelialization. From HE staining (Figure 8C), this study found that all wounds were completely covered with regenerated epithelial cells after 10 days, while epithelial cells proliferated, differentiated, and transformed for strength enhancement for complete wound healing. The length of newly formed epithelium, together with average thicknesses for wound granulation tissue, was assessed from the HE staining. From Figure 8D,E, the length of regenerated epithelium considerably decreased within OBC‐pVEs group (1105.23 ± 115.55 µm) compared with the control (1423.91 ± 71.32 µm), gauze (1400.67 ± 38.77 µm), and OBC group (1336.06 ± 20.58 µm) (*p* = 0.003, *p* = 0.005, *p* = 0.021). However, there was no significant difference between OBC‐pVEs and OBC‐DA groups (1105.23 ± 115.55 µm versus 1139.29 ± 30.85 µm, *p* = 0.716). The wound granulation tissue thickness of OBC‐pVEs was decreased significant with gauze (471.90 ± 20.41 µm versus 716.77 ± 64.62 µm, *p* = 0.022) and OBC (668.30 ± 60.06 µm, *p* = 0.03), but no significant with control (644.98 ± 104.01 µm, *p* = 0.094) and OBC‐DA (701.54 ± 72.29 µm, *p* = 0.739). Granulation tissue maturation was evaluated histologically 10 days after inflicting wounds by measuring granulation collagen deposition. Extensive collagen deposition, indicating recovery and regeneration of damaged tissues, was observed in OBC‐pVEs aerogel‐treated wounds (*p* < 0.001) (Figure 8F,G). These results further confirmed that OBC‐pVEs aerogel can enhance wound healing and re‐epithelialization.

### 2.9 OBC‐pVEs Promoted Re‐Epithelialization and Angiogenesis

**Figure 9:**
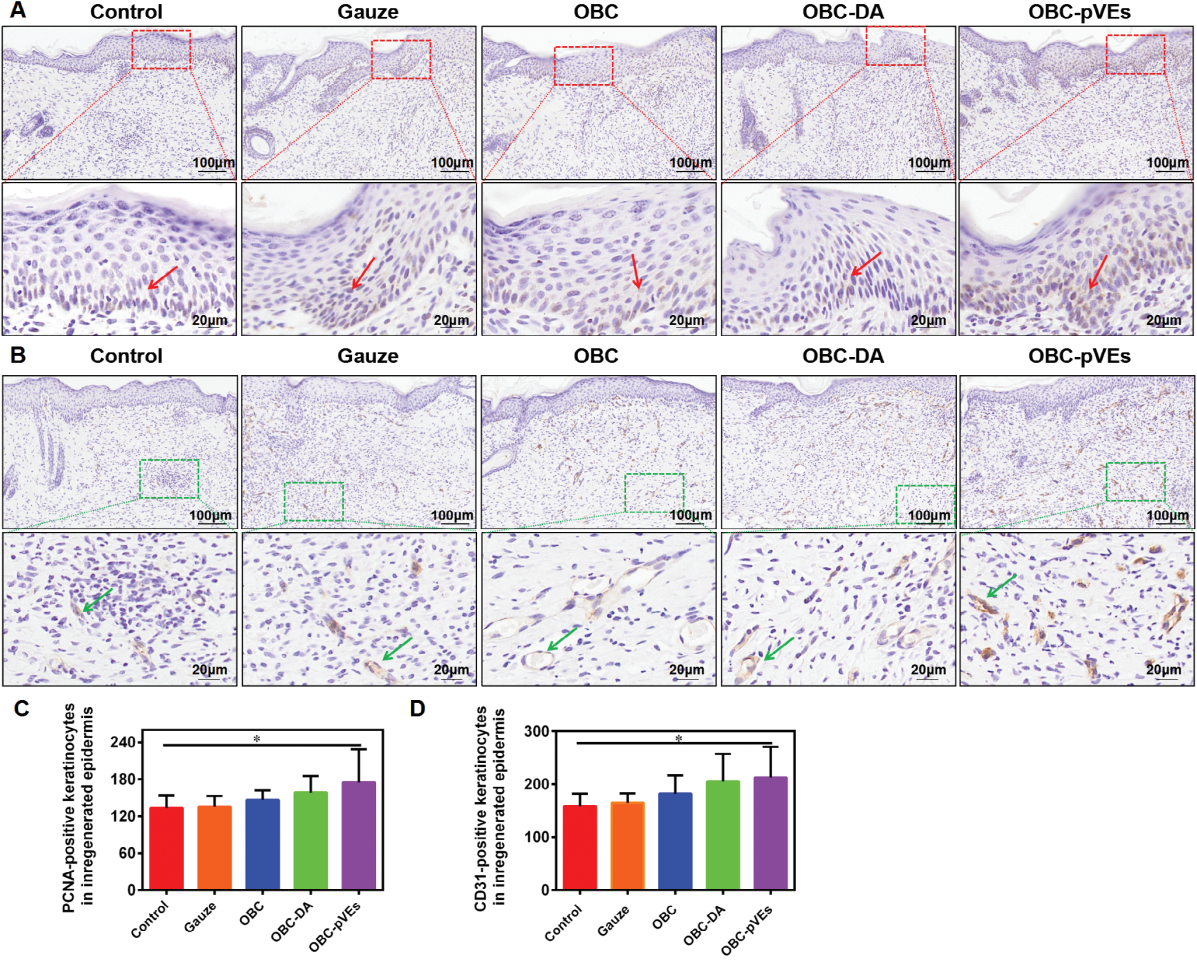
PCNA, CD31 immunohistochemical staining. A) Expression of PCNA within wound tissue. B) Expression of CD31 within wound tissue. C) PCNA‐positive cell population within wound at the day 10. D) Density for blood vessels within the wound at day 10. In (C) and (D), datasets reflect means ± SD (*n* = 5, One‐way ANOVA, *p < 0.05). The red arrows indicated proliferating cells, and the green arrows indicated angiogenesis.

Promoted re‐epithelialization could be discerned through keratinocyte proliferative/migrative properties. Angiogenesis is also an important indicator of wound healing. PCNA, a biomarker for cell proliferative property, and CD31, a biomarker for angiogenesis,^[^ ^31^ ^]^ were detected by immunohistochemistry (**Figure** **9**A,B). PCNA‐positive keratinocytes across wound boundaries in the OBC‐pVEs group were considerably elevated in comparison to the control group at day 10 (Figure 9C,D, P < 0.05). OBC‐pVEs significantly increased blood vessel density compared to the control group (P < 0.05). Thus, regenerated epidermis development/angiogenesis promotes and accelerates rapid skin wound closure due to the bioactivity, cytocompatibility, and histocompatibility of OBC‐pVEs. Taken together, data presented in this study strongly suggested such developed OBC‐pVEs aerogels can be effective for hemostasis and wound dressings, and may have the potential for novel therapeutics against cutaneous wounds.

## 3 Conclusion

In this work, we developed OBC‐pVEs aerogels as injectable shape memory hemostatic bioactive dressings as rapidly acting and efficient hemostatic materials to treat deep, non‐compressible, and irregular wounds. In vivo hemostasis tests, including rat liver and mouse tail hemorrhage models, demonstrated that the OBC‐pVEs could achieve hemostasis fast and decrease blood loss. The excellent hemostasis is attributed to effective blood clotting capacity, high blood cell adsorption capacity, enrichment of coagulation factors, and platelet activation together with fibrin formation. OBC‐pVEs aerogel can also accelerate wound healing in full‐thickness skin defects. Therefore, OBC‐pVEs aerogels provide an effective tool for treating deep, non‐compressible, and irregular wounds.

## 4 Experimental Section

### Materials

Peptone, yeast extract, and agar powder were obtained from OXOID. NaClO, NaBr, 2,2,6,6‐tetramethylpiperidine‐1‐oxyl (TEMPO), and citrate‐phosphate‐dextrose buffer were obtained from Sigma‐Aldrich. Absorbable Gelatin Sponge (AGS) (Jinling Pharmaceutical Company Limited), dopamine hydrochloride (Aladdin DA‐HCL, China), cell counting kit‐8 (CCK‐8) from Dojindo Laboratories (Kumamoto, Japan), CD3, F4/80 antibodies (BioLegend; California, USA), CD63, Alix, TSG101, PCNA, CD31 antibodies (CST; Boston, USA), and *Gluconacetobacter xylinum* (ATCC) were used as received. All in vivo studies were performed with approval from the Institutional Animal Care and Use Committee for Third Military Medical University (AMUWEC20224151, Army Medical University).

### Preparation and Characterization of BC Membrane

BC membranes were produced by incubation of *G. xylinum* in Hestrin and Schramm (HS) growth medium containing glucose (25 g L^−1^), yeast extract (5 g L^−1^), peptone (5 g L^−1^), KH_2_PO_4_ (0.75 g L^−1^), MgSO_4_ (0.2 g L^−1^) and citric acid (1.15 g L^−1^). The medium was autoclaved at 15 psi and 121 °C for 30 min before use, and the pH was adjusted to 5.5. *G. xylinum* culture plate colonies were inoculated in 150 mL HS broth in a 250 mL Erlenmeyer flask and incubated for two days at 30 °C and 160 rpm). From this pre‐culture, 6% v/v was inoculated into fresh 1L HS medium (pH 5.5) and incubated statically at 30 °C for 10 days. The resulting BC sheets were harvested and treated with 0.1 m NaOH (20 min; 100 °C) to disrupt and dissolve cellular debris. BC sheets were then rinsed using deionized and distilled water until the pH reached 7.^[^ ^32^ ^]^ Finally, the BC membranes were treated with a high‐speed blender, followed by freeze‐drying. The membranes’ morphology was visualized with scanning electron microscopy (SEM, SU8020, HITACH, Japan).

### TEMPO‐Mediated Oxidation of BC Nanofibers and Preparation of OBC Aerogels

Bacterial cellulose nanofibers (1 g) were placed into 100 mL aqueous suspension containing TEMPO (0.016 g, 0.1 mmol) and sodium bromide (0.1 g, 1 mmol). NaClO solution (5.0 mmol NaClO per gram of BC nanofibers) was added, and the oxidation proceeded at ambient temperature under stirring (500 rpm). The pH was adjusted to 10 using 0.5 m NaOH.^[^ ^33^ ^]^ The TEMPO‐oxidized cellulose was thoroughly washed with water and re‐suspended in water at 0.2%, 0.4%, 0.6%, 0.8%, 1.0%, and 1.2% concentrations. OBC aerogels were prepared by cyclic freezing‐thawing (FT) different concentrations of OBC suspensions. OBC suspension (2 mL) of various concentrations was put into each well of a 24‐well plate or 1 mL into a 48‐well plate. Cyclic freezing‐thawing was conducted at −20 °C for 15 h and then thawing at ambient temperature for 9 h as one FT cycle. After three FT cycles, hydrogels were placed into tert‐butanol to exchange water for 12 h three times, and then freeze‐dried to obtain OBC aerogels.^[^ ^26^ ^]^ The height and water absorption of OBC with different concentrations were measured. The porosity of OBC aerogels was characterized by Mercury Intrusion Porosimetry (Autopore IV 9620, Micromeritics, USA). The rapid shape recovery of OBC aerogels after water absorption was recorded by digital imaging. The mechanical and rheological properties were tested by a universal testing machine (MTS) and a rheometer (DHR‐1, TA), respectively.

### Extraction and Characterization of pVEs

Sprague‐Dawley (SD) rat platelets were isolated from whole blood by gradient centrifugation as previously described.^[^ ^21^ ^]^ Briefly, platelets 1 mL rat whole blood was mixed with 0.8 mL of citrate‐phosphate‐dextrose buffer and centrifuged at 100 × g for 20 min at 25 °C. Afterwards, the supernatant was centrifuged at 800 × g for 10 min at 25 °C and suspended in 0.5 mL hypotonic lysis buffer (20 mM Tris‐HCl, 10 mM KCl, and 2 mM MgCl_2_). The platelets were sonicated using a Digital Sonifier 450 (Branson Ultrasonics, USA). The condition of sonication was 30% power for 40 s (20 s pulse on and 20 s pulse off). The solution was centrifuged at 16000 × g for 20 min at 4 °C, the pellet was discarded, and the supernatant was stored at 4 °C for further use.^[^ ^24^ ^]^ Transmission electron microscope (TEM; JEOL, Japan) images of pVEs were obtained by placing them on 400 mesh copper grids and staining them with a 2% uranyl acetate solution. A dynamic light scattering (DLS) particle size analyzer (Malvern 2000, USA) was used to determine the hydrodynamic diameters and zeta potential of the pVEs, while the movement of sample particles was detected through NTA (Nanoparticles Tracking Analysis, Particle Metrix, Germany). Identification of the pVEs for CD63, TSG101 and Alix biomarkers was performed by Western blotting analysis. Mice were anesthetized, and the end of each tail (2 mm) was fully transected. Tails were placed on filter paper, and bleeding was monitored. Where the mice were given 100 µL pVEs or equal volume saline (control group) via tail vein injection 30 min before starting the tail bleed assay, blood loss was quantified (mg) .^[^ ^24^ ^]^

### Preparation and Characterization of OBC‐pVEs Aerogels

OBC (0.8 wt%) aerogels were immersed in polydopamine (DA) solution (2 mg mL^−1^ in 10 mM Tris‐HCL, pH 8.5) at 4 °C for 16 h. Then, the specimens were washed with water three times to remove the residual DA, and the DA‐coated OBC gels were immersed in pVEs solution (800 µL) for 24 h in the dark at 4 °C. Finally, OBC‐DA and OBC‐pVEs aerogels were obtained by freeze‐drying. The OBC, OBC‐DA, and OBC‐pVEs aerogels were observed using SEM, and the porosity, specific surface area, and bulk density were measured by Mercury Intrusion Porosimetry. Mechanical and rheological properties were tested according to the same methods as described earlier, and the process of the OBC‐pVEs aerogels with rapid shape recovery after water absorption was recorded by a digital camera.

### In Vitro Biocompatibility Evaluation of OBC‐pVEs Aerogels

The cytotoxicity of OBC‐pVEs aerogels was determined by CCK‐8 assay in vitro.^[^ ^34^ ^]^ Briefly, 3T3 cells were seeded into a 96‐well culture plate at a density of 2×10^3^ cells per well and incubated at 37 °C in an incubator with 5% CO_2_ for 24 h. Following this, the cell culture medium was aspirated, and fresh culture media containing extracts of the different groups of aerogels were added. Cells were then gently washed once with sterile PBS and then treated with 100 µL fresh culture medium and 10 µL CCK‐8 solution and further incubated at 37 °C for 2 h. The cell viability was then quantified by measuring the absorbance at 450 nm using a Microplate reader (Thermo Varioskan Flash, USA), and the cell morphology was observed by fluorescence staining. The hemolysis assay was performed using a previously reported method.^[^ ^35^ ^]^ Fresh whole blood samples were collected from the orbital venous of healthy SD rats. OBC, OBC‐DA, and OBC‐pVEs aerogels were incubated in a water bath at 37 °C for 30 min with 5 mL normal saline, and then 200 µL whole blood was added and incubated at 37 °C for 2 h. The normal saline was used as the negative control and distilled water was used as the positive control. The tubes were centrifuged for 10 min at 1500 rpm, and then the optical density of the supernatant fluid was read by a Microplate reader at 545 nm. The hemolysis rate (HR) was calculated according to the following formula:

(1) $$\mathrm{HR}\%=\left[\mathrm{ODs}-\mathrm{ODn}\right]/\left[\mathrm{ODp}-\mathrm{ODn}\right]\times 100\%$$

ODs, ODn, ODp are the corresponding optical density (OD) values of the sample, negative control and positive control groups, respectively. Experiments were repeated three times for each group.

### In Vitro Blood Coagulation Evaluation of OBC‐pVEs Aerogels

A coagulation assay was performed according to a previously reported protocol.^[^ ^36^ ^]^ Briefly, fresh whole blood was collected from the orbital venous of healthy SD rats in citric acid anticoagulant tubes. AGS, OBC, OBC‐DA, OBC‐pVEs samples were added to 24‐well plates. Then, 100 µL of the anticoagulated whole blood was added into the wells, followed by 2 mL of distilled water after 2 min. The liquid was immediately aspirated and read by a Micro‐plate reader at 545 nm. For SEM, samples were fixed in 2% glutaraldehyde for 12 h, gradient‐dehydrated in ethanol and tert‐butanol, sputter‐coated with gold, and then examined by SEM. The coagulation rate was calculated according to the following formula:

(2) $$\mathrm{CR}\%=\left[\mathrm{ODs}-\mathrm{ODn}\right]/\left[\mathrm{ODp}-\mathrm{ODn}\right]\times 100\%$$

ODs, ODn, ODp are the corresponding OD values of the sample, negative control, and positive control groups, respectively, and three repeats were conducted for each group.

### In Vivo Hemostatic Ability

All experimental animals were purchased from the Experimental Animal Center of the Third Military Medical University. A rat hemorrhaging liver model (male SD rats, 200 g) and a mouse tail amputation model (male BALB/c mice, 20 g) were used to investigate the in vivo hemostatic ability of OBC‐pVEs aerogels.^[^ ^37^ ^]^ The aerogels (with a diameter of 15 mm and height of 6 mm) were hydrated with water and then loaded into a syringe for in vivo injection. For the rat hemorrhaging liver model, rats were anesthetized by 1% pentobarbital. Then, the liver was punctured to bleed with a puncture needle (diameter of 6 mm, depth 3 mm). AGS could not be injected with a syringe, so it was directly placed on the wound surface. A pre‐weighed filter paper was placed beneath the liver. Immediately, the aerogel was injected into the wound site as a hemostatic agent. The weight of the filter paper with blood was measured, and the mass of blood was calculated until the bleeding stopped (at least five min). The hemostatic times of AGS treated and blank control groups were measured. For the mouse tail amputation model, mice were anesthetized by 1% pentobarbital. Then, a pre‐weighed filter paper was placed beneath the tail, and the tail was cut 2 cm away from the base. Immediately, the aerogel was put onto the bleeding site for a certain time. The mass of blood was measured until the bleeding stopped. The hemostatic times of AGS treated and blank groups were measured.^[^ ^14^ ^]^

### In Vivo Biocompatibility Evaluation and Degradation of OBC‐pVEs Aerogels

To evaluate the biocompatibility and degradation of OBC‐pVEs aerogels in vivo, BALB/c mice (male, aged 8 weeks, 20 g) were anesthetized with 1% pentobarbital, and a 1cm‐long incision was created. The sterilized OBC‐pVEs aerogel was implanted. On days 3, 7, 14, 21, and 28, the mice were euthanized, and the remaining OBC‐pVEs aerogels were weighed.^[^ ^38^ ^]^ The inflammatory response was analyzed by H&E and immunofluorescence stainings.

### In Vivo Therapeutic Effect of OBC‐pVEs Aerogels on Wound Healing

Briefly, male BALB/c mice (aged 8 weeks, 20 g, n = 3) were intraperitoneally injected with 1% pentobarbital to be anesthetized. Full‐thickness wounds were simultaneously created in the dorsal skin using a sterile 6‐mm diameter punch. 6 mm diameter silicone ring was held on each wound to represent the initial wound area, and the wounds were photographed immediately using a digital camera.^[^ ^39^ ^]^ Following this, each wound in the treatment group was covered with the aerogels. Wounds in the control group were left untreated while the gauze group was covered with gauze. Then, the wounds were covered with a piece of Tegaderm Transparent Film (3 M, China). The wounds were photographed on days 3, 5, 7, and 10 post‐surgery.^[^ ^40^ ^]^ Wound areas were measured using ImageJ software. The wound healing rate was calculated based on the following formula:

(3) $$\mathrm{Wound}\;\mathrm{healing}\;\mathrm{rate}\;\%=\left(I-R\right)/I\times 100\%$$

where I represents the initial wound area, and R represents the remaining wound area on the determined day post‐surgery.

At day 10 post‐surgery, mice were sacrificed to harvest wound tissues for histological analysis. The wound tissues were fixed with 4% paraformaldehyde and embedded in paraffin for H&E staining and Masson staining and detected by immunohistochemistry for PCNA antibody (13110S, 1:4000 dilution) and CD31 antibody (77699S, 1:500 dilution). The length of the regenerated epidermis and the thickness of granulation tissues and collagen deposition were quantified using Image J software (v1.8.0).

### Statistical Analysis

Data are presented as means ± SD of at least three independent measurements using GraphPad Prism 6.0 (GraphPad Software, USA). Statistical analyses were performed by two‐tailed Student's *t*‐test or one‐way ANOVA using SPSS 20.0 (IBM, USA). Tukey's post hoc test was used for multiple post hoc comparisons to determine statistical significance. *p* < 0.05 was considered significant.

## Conflict of Interest

The authors declare no conflict of interest.

## Supporting information

Supporting Information

Supplemental Movie 1

Supplemental Movie 2

Supplemental Movie 3

Supplemental Movie 4

Supplemental Movie 5

## Data Availability

The data that support the findings of this study are available from the corresponding author upon reasonable request.
