# Supplementary material for: Platelet Vesicles Synergetic with Biosynthetic Cellulose Aerogels for Ultra‐Fast Hemostasis and Wound Healing
Source: Adv Healthc Mater. 2024 Feb 28;13(17):2304523. doi: 10.1002/adhm.202304523 (PMC11469313; doi:10.1002/adhm.202304523)
Supplement: Supplementary file 1 — Supporting Information [file ADHM-13-2304523-s005.pdf]

ADVANCED HEALTHCARE MATERIALS

Supporting Information

for *Adv. Healthcare Mater.*, DOI 10.1002/adhm.202304523

Platelet Vesicles Synergetic with Biosynthetic Cellulose Aerogels for Ultra-Fast Hemostasis and Wound Healing

Ying Wang, Yicheng Guo, Yuqing Liu, Xiaohong Zhao, Yong Huang, Xiaorong Zhang, Xiaohong Hu, Kibret Mequanint, Gaoxing Luo and Malcolm Xing**

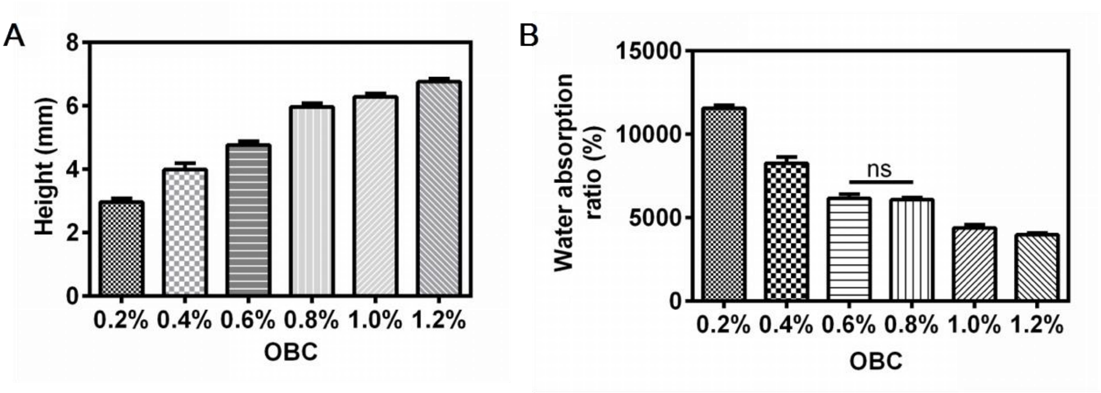

Figure. S1. (A) The height chart of 0.2 ~ 1.2 wt% OBC aerogel (N = 5). (B) The water absorption radio of 0.2 ~ 1.2 wt% OBC aerogel (N = 3).

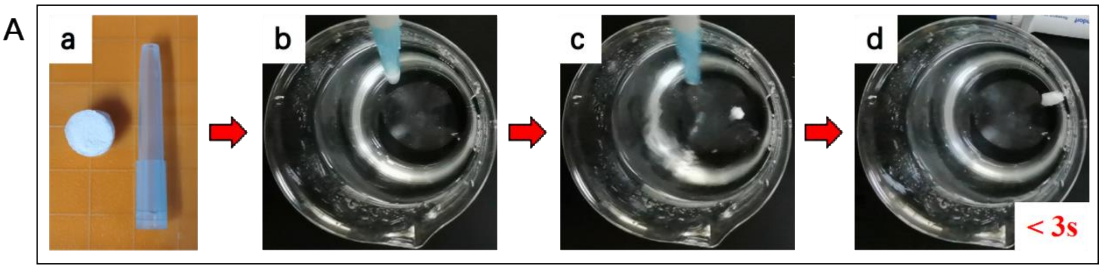

Figure. S2. (A) 0.8% OBC aerogel: (a) in air, (b) Load into 1ml pipette tip after wetting, (c) Shot out from the pipette tip, (d) sequential snapshots of (c) reabsorbing water to full shape recovery in 3 s.

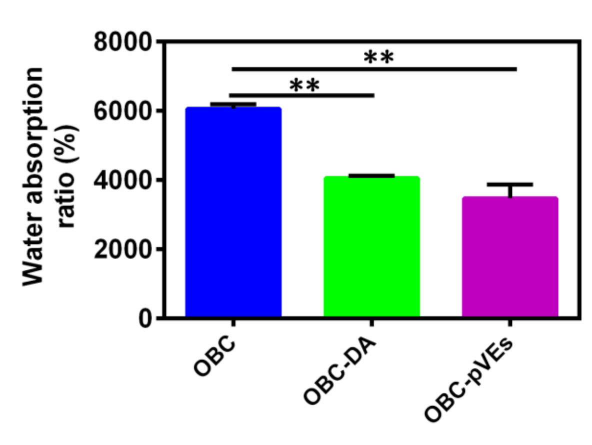

Figure. S3. The water absorption radio of OBC, OBC-DA and OBC-pVEs aerogel (N = 3, One-way ANOVA, **P < 0.05).

Table S1 Mercury Intrusion Porosimetry test results of 0.2 ~ 1.2 wt% OBC aerogels

Samples	0.2%	0.4%	0.6%	0.8%	1.0%	1.2%

**Porosity（%）**	91.91±0.43	91.68±1.59	93.25±0.70	97.35±0.36	89.92±2.42	92.56±0.45
**Total pore area (m^2/g)**	12.81±5.99	19.21±4.26	23.13±8.98	42.22±8.21	42.23±10.31	39.57±8.34
**Density （g/mL）**	0.020±0.002	0.023±0.001	0.029±0.006	0.037±0.005	0.044±0.005	0.050±0.012
**Pore size (μm)**	28161±9579	11900±1494	7660±384	6292±307	4813±414	2555±420

Table S2 Mercury Intrusion Porosimetry test results of OBC, OBC-DA and OBC-pVEs aerogels

**Samples**	**OBC**	**OBC-DA**	**OBC-pVEs**
**Porosity（%）**	97.35±0.36	93.14±2.61	91.46±0.60
**Total pore area (m^2/g)**	42.22±8.21	2.81±0.27	1.79±0.15
**Density（g/mL）**	0.037±0.005	0.047±0.007	0.062±0.011

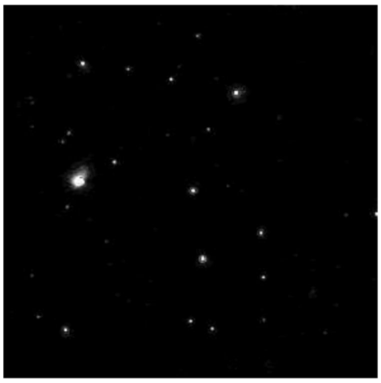

Figure. S4. NTA result showing the pVEs particle movement.
